# Supplementary material for: Boosting test-efficiency by pooled testing for SARS-CoV-2—Formula for optimal pool size
Source: PLoS One. 2020 Nov 4;15(11):e0240652. doi: 10.1371/journal.pone.0240652 (PMC7641378; doi:10.1371/journal.pone.0240652)
Supplement: S1 File — (PDF) [file pone.0240652.s001.pdf]

S1 File

Boosting test-efficiency by pooled testing for SARS-CoV-2 – formula for optimal pool size"

Rudolf Hanel[1,2], Stefan Thurner[1,2,3,4],

1 Section for Science of Complex Systems, Medical University of Vienna, Spitalgasse 23, 1090 Vienna, Austria **2** Complexity Science Hub Vienna, Josefstädterstrasse 39, 1080 Vienna, Austria **3** Santa Fe Institute, 1399 Hyde Park Road, Santa Fe, NM 87501, USA **4** IIASA, Schlossplatz 1, 2361 Laxenburg, Austria

* To whom correspondence should be addressed. E-mail: stefan.thurner@muw.ac.at

SI1 Derivation of optimal group size

We recall that the probability of a randomly sampled group of ω individuals to have at least one infected in the group is given by $p = 1 - (1-\lambda)^\omega$, where λ is the disease prevalence, i.e. the fraction of infected in the general or considered population. Also, the number of persons per test PPT is given by $1/Q$, where the expected numbers of tests per person, Q, is given in Eq. (5) of the main document, and therefore we get

$$PPT = \frac{1}{P_+^* + \frac{r}{\omega}} \,, \tag{SI1.1}$$

where $P_+^* = p(1-\gamma_+^* - \gamma_-^*) + \gamma_+^*$ (compare main document Eq. (4)). γ_+^* and γ_-^* are the effective false negative and false positive rates of pooled tests with multiple replicates, given in Eq. (2) and Eq. (3) of the main document. We note that neither γ_+^* nor γ_-^* depend on ω and we can compute

$$\frac{d}{d\omega} PPT = -PPT^2 \left(-\log(1-\lambda)(1-\lambda)^\omega (1-\gamma_+^* - \gamma_-^*) - \frac{r}{\omega^2} \right) \,. \tag{SI1.2}$$

For obtaining the extremal ω, we have to solve $\frac{d}{d\omega} PPT = 0$. This implies

$$\omega^2 e^{\log(1-\lambda)\omega} = \frac{r}{(1-\gamma_+^* - \gamma_-^*)\log\left(\frac{1}{1-\lambda}\right)} \,, \tag{SI1.3}$$

and further,

$$\frac{1}{2}\log(1-\lambda)\omega e^{\frac{1}{2}\log(1-\lambda)\omega} = \frac{1}{2}\log(1-\lambda)\left(\frac{r}{(1-\gamma_+^* - \gamma_-^*)\log\left(\frac{1}{1-\lambda}\right)}\right)^{\frac{1}{2}} \,. \tag{SI1.4}$$

As a consequence we obtain

$$\omega^{\rm opt} = \frac{W_0\left(\frac{1}{2}\log(1-\lambda)\left(\frac{r}{(1-\gamma_+^* - \gamma_-^*)\log\left(\frac{1}{1-\lambda}\right)}\right)^{\frac{1}{2}}\right)}{\frac{1}{2}\log(1-\lambda)} \,, \tag{SI1.5}$$

where W_0 is the principal branch of the Lambert-W function. For small λ, using that $\log(1/(1-\lambda)) \sim \lambda$, and that $W_0(x) \sim x$ for $|x| \ll 1$, this approximately yields

$$\omega^{\text{opt}} \sim \left(\frac{r}{(1-\gamma_+^* - \gamma_-^*)\lambda} \right)^{\frac{1}{2}} . \qquad \text{(SI1.6)}$$

SI2 Majority rules and dependency on numbers of replicates

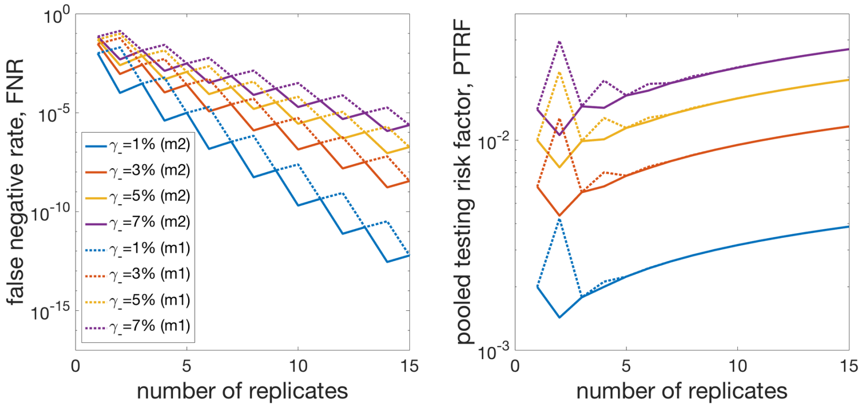

Fig SI2.1. Majority rule type and replicates. (A) *False negative rate* (FNR) are displayed for majority rule (m1) in dotted lines and (m2) in full lines, for false negative rates of $\gamma_- = 0.01$, 0.03, 0.05, and 0.07. (B) *Pooled testing risk factor* (PTRF) are also displayed for majority rule (m1) in dotted lines and (m2) in full lines, for false negative rates of $\gamma_- = 0.01$, 0.03, 0.05, and 0.07.

In Fig. SI2.1 we see that both, the *false negative rate*, FNR, of pooled tests with multiple replicates, which is not group size dependent, and the respective *pooled testing risk factor*, PTRF, given at the optimal group size behave differently in the sense that over all we can observe that FNR decreases (compare Fig. SI2.1A) with growing numbers of replicates while PTRF increases (compare Fig. SI2.1B) . However, FNR is not decreasing strictly monotonically. For majority rule m1, that a group is considered positive if there more replicate tests have registered positive than negative, all even numbers, $2n$, of replicates yield higher FNR than for the adjacent odd numbers of replicates $2n+1$ and $2n-1$. For majority rule m2, that a group is considered positive if there are at least as many replicate tests that have registered positive as replicates that have registered negative, the situation is reversed. All even numbers, $2n$, of replicates yield a lower FNR than respective tests with odd numbers of replicates $2n+1$ and $2n-1$. Similarly, for the *pooled testing risk factor*, PTRF, tests with even numbers of replicates also yield consistently lower PTRF values for rule m2 than rule m1 (compare Fig. SI2.1B). However, the gain in PTRF is most pronounced for two replicates, while for numbers of replicates larger than 5, there remains hardly any observable difference between the two versions of the majority rule. As a consequence we can safely conclude that rule m2 is consistently superior to rule m1 with respect to controlling false negative rates related measures such as FNR and PTRF.

SI3 Matlab Code: single_stage_group_test.m

Here we provide the code for a Matlab function (single_stage_group_test.m), which can be used to reproduce all results and plots produced in the main paper. The input variables are explained in the header of the m-file. The functions output is a Matlab struct `out` with items

- `out.PPT` ... *persons per test* values given as a $N_r \times \Omega_m ax$-matrix for N_r numbers of replicates (e.g. setting variable $Rv = [1, 2, 3, 4, 5]$ means $N_r = 5$)
- `out.PTRF` ... *pooled testing risk factor* also a $N_r \times \Omega_m ax$-matrix
- `out.oPPT` ... PPT values; vector of length N_r holding the values of PPT for the optimal group sizes provided in the input variable Rv .
- `out.oPTRF` ... PTRF values; vector of length N_r holding the values of PTRF for the optimal group sizes with respect to the numbers of replicates provided in the input variable Rv .

```
%%%%%%%%%%%%%%%%%%%%%%%%%%%%%%%%%%%%%%%%%%%%%%
% variables:
%%%%%%%%%%%%%%%%%%%%%%%%%%%%%%%%%%%%%%%%%%%%%%
% fposv ... (1) the const false positive rate
%           (template 0.014) or
%           (2) [fpos1, fpos2] fpos will be
%           linearly interpolated between
%           omega=1 (fpos1) and poolsize_max (fpos2)
% fnegv ... (1) the const false negative rate
%           (template 0.02) or
%           (2) [fneg1, fneg2] fneg will be
%           linearly interpolated between
%           omega=1 (fneg1) and poolsize_max (fneg2)
% poolsize_max ... the maximally considered pool
%           size (template value 100)
% lambda ... the infection level (dissease prevalence)
%           as the fraction of the infected
%           population (not percent!!!)
% Rv ...    number of replicas (can be a vector)
% is_m2 ... if true -> majority rule m2: multiple
%           replicas are positive if at least as
%           many positives as negatives
%           if false -> majority rule m1: more
%           replicas need to bepositive than negative
%           for the replicated pooled testto be positive
% plotflg ... if true the out put variable (PPT, PTRF)
%           get plotted into a new figure
%%%%%%%%%%%%%%%%%%%%%%%%%%%%%%%%%%%%%%%%%%%%%%
function out = single_stage_group_test(fposv,fnegv,poolsize_max
    ,lambda,Rv,is_m2,plotflg);
%%%%%%%%%%%%%%%%%%%%%%%%%%%%%%%%%%%%%%%%%%%%%%%
% templates and variable handling
if length(fposv)==0,
    fposv=0.014;
end;
```

```
if length(fnegv)==0,
     fposv=0.02;
end;
if length(fposv)==1;
     fposv=[fposv fposv];
end;
if length(fnegv)==1;
     fnegv=[fnegv fnegv];
end;
if length(poolsize_max)==0,
     poolsize_max=100;
end;
if length(lambda)==0,
     lambda=0.01;
end;
if length(is_m2)==0,
     is_m2=true;
end;
if length(plotflg)==0,
     plotflg=true;
end;
%%%%%%%%%%%%%%%%%%%%%%%%%%%%%%%%%%%%%%%%
% remember variables
isgeq=is_m2;
out.poolsize_max=poolsize_max;
out.lambda=lambda;
out.R=Rv;
out.is_m2=isgeq;
out.is_m1=~(isgeq);
out.fpos=fposv;
out.fneg=fnegv;
%%%%%%%%%%%%%%%%%%%%%%%%%%%%%%%%%%%%%%%%
% compute ...
%%%%%%%%%%%%%%%%%%%%%%%%%%%%%%%%%%%%%%%%
NR=length(Rv);
%%%%%%%%%%%%%%%%%%%%%%%%%%%%%%%%%%%%%%%%
% const for linear increasing fn with poolsize
fpnv=0:(poolsize_max-1);
%%%%%%%%%%%%%%%%%%%%%%%%%%%%%%%%%%%%%%%%
fpos1=fposv(1);
fposfac=fposv(2)/fposv(1);
fpinc=(fposfac-1)/(poolsize_max-1);
fpfacv=1+fpnv*fpinc;

fneg1=fnegv(1);
fnegfac=fnegv(2)/fnegv(1);
fninc=(fnegfac-1)/(poolsize_max-1);
fnfacv=1+fpnv*fninc;
%%%%%%%%%%%%%%%%%%%%%%%%%%%%%%%%%%%%%%%%
% lambda ... probability to have an infected person in
%            a population of N
%%%%%%%%%%%%%%%%%%%%%%%%%%%%%%%%%%%%%%%%
```

```
%%%%%%%%%%%%%%%%%%%%%%%%%%%%%%%%%%%%%%%%%%%%%%
% constants ...
%%%%%%%%%%%%%%%%%%%%%%%%%%%%%%%%%%%%%%%%%%%%%%
% for switching from greater or equal than to greater than
% depending on the majority protocol (0<eps<1)
eps=0.1;
%%%%%%%%%%%%%%%%%%%%%%%%%%%%%%%%%%%%%%%%%%%%%%
%%%%%%%%%%%%%%%%%%%%%%%%%%%%%%%%%%%%%%%%%%%%%%
% expected number of persons per test (PPT)
out.PPT=zeros(NR,poolsize_max);
% expected maximal number of missed positive individuals (PTRF)
out.PTRF=zeros(NR,poolsize_max);
% optimal group size
out.oGS=zeros(NR);
% values at optimal group size
out.oPPT=zeros(NR);
out.oPTRF=zeros(NR);
%%%%%%%%%%%%%%%%%%%%%%%%%%%%%%%%%%%%%%%%%%%%%%
%%%%%%%%%%%%%%%%%%%%%%%%%%%%%%%%%%%%%%%%%%%%%%
% loop over pool size
for omega=1:poolsize_max,
    fpos=fpos1*fpfacv(omega);
    fneg=fneg1*fnfacv(omega);
    %%%%%%%%%%%%%%%%%%%%%%%%%%%%%%%%%%%%%%%%%%
    % loop over replicates
    for rid=1:NR,
        R=Rv(rid);
        %%%%%%%%%%%%%%%%%%%%%%%%%%%%%%%%%%%%%%
        % switch from greater or equal than to greater than
        % depending on the majority protocol
        if isgeq,
            peps=0;
            neps=eps;
        else
            peps=eps;
            neps=0;
        end;
        %%%%%%%%%%%%%%%%%%%%%%%%%%%%%%%%%%%%%%
        % probability that at least one individual in the pool is positive
        ppos=(1-(1-lambda)^omega);
        %%%%%%%%%%%%%%%%%%%%%%%%%%%%%%%%%%%%%%
        % compute the false positive and false negative rate for replica
        % the probability that the majority (m1 or m2 depending on geq)
        % of R tests is falsly positive
        fpeff=0;
        for n=ceil((R+peps)/2):R,
            fpeff=fpeff+nchoosek(R,n)*(fpos^n)*((1-fpos)^(R-n));
        end;
```

```
        % the probability that we miss a positive groupe
        fneff=0;
        for n=ceil((R+neps)/2):R,
            fneff=fneff+nchoosek(R,n)*(fneg^n)*((1-fneg)^(R-n));
        end;
        %%%%%%%%%%%%%%%%%%%%%%%%%%%%%%%%%%%%%%
        % prob of positive pool
        ppp=ppos*(1-fpeff-fneff)+fpeff;
        %%%%%%%%%%%%%%%%%%%%%%%%%%%%%%%%%%%%%%
        R_id=rid;
        if (omega>=R),
            out.PPT(R_id,omega)=1./(ppp+R/omega);
            out.PTRF(R_id,omega)=ppos*(fneff+(1-fneff)*fneg);
        else
            out.PPT(R_id,omega)=NaN;
            out.PTRF(R_id,omega)=NaN;
        end;
        %%%%%%%%%%%%%%%%%%%%%%%%%%%%%%%%%%%%%%
    end;
end;
%%%%%%%%%%%%%%%%%%%%%%%%%%%%%%%%%%%%%%%%%%%%%%
%%%%%%%%%%%%%%%%%%%%%%%%%%%%%%%%%%%%%%%%%%%%%%
for rid2=1:NR,
    v=find(out.PPT(rid2,:)==max(out.PPT(rid2,:)));
    xid=min(100,v(1));
    if (lambda>0.01 && xid==100),
        out.oGS(rid2)=NaN;
        out.oPPT(rid2)=NaN;
        out.oPTRF(rid2)=NaN;
    else
        out.oGS(rid2)=xid;
        out.oPPT(rid2)=out.PPT(rid2,xid);
        out.oPTRF(rid2)=out.PTRF(rid2,xid);
    end;
end;
%%%%%%%%%%%%%%%%%%%%%%%%%%%%%%%%%%%%%%%%%%%%%%
%%%%%%%%%%%%%%%%%%%%%%%%%%%%%%%%%%%%%%%%%%%%%%

if plotflg,
    figure;
    FS=26;
    subplot(1,3,1);
    h1=plot(out.PPT','LineWidth', 3);
    ax = gca;
    ax.FontSize = 24;
    xlabel('pool size, \omega','FontSize',FS);
    ylabel('PPT','FontSize',FS);
    subplot(1,3,2);
    h2=plot(out.PTRF','LineWidth', 3);,
    %axis([0 100 0 1])
    ax = gca;
```

```
    ax.FontSize = 24;
    xlabel('pool size, \omega','FontSize',FS);
    ylabel('PTRF', 'FontSize',FS);
    subplot(1,3,3);
%    h3=plot(out.PPT',out.PTRF','LineWidth', 3);,
    h3=plot(out.PTRF',out.PPT','LineWidth', 3);,
    %axis([0 100 0 1])
    ax = gca;
    ax.FontSize = 24;
    ylabel('PPT','FontSize',FS);
    xlabel('PTRF', 'FontSize',FS);
end;

%%%%%%%%%%%%%%%%%%%%%%%%%%%%%%%%%%%%%%%%
% END
%%%%%%%%%%%%%%%%%%%%%%%%%%%%%%%%%%%%%%%%
```

SI4 Matlab files

In the zipped SI file we also provide the following m-files. Three scripts that we used for producing the three figures in the main document and the figure in SI2. For convenience, we also provide the code above as a Matlab function file.

- single_stage_group_testing_fig1.m (script)
- single_stage_group_testing_fig2.m (script)
- single_stage_group_testing_fig3.m (script)
- single_stage_group_testing_figSI1.m (script)
- single_stage_group_test.m (the function printed above)
