# Supplementary material for: Boosting test-efficiency by pooled testing for SARS-CoV-2—Formula for optimal pool size
Source: PLoS One. 2020 Nov 4;15(11):e0240652. doi: 10.1371/journal.pone.0240652 (PMC7641378; doi:10.1371/journal.pone.0240652)
Supplement: Attachment — Submitted filename: Response to Reviewers 14.08.2020.pdf [file pone.0240652.s003.pdf]

Vienna, Aug 11, 2020

To the
PLOS ONE Editorial Board

Subject: Resubmission of the manuscript *Boosting test-efficiency by pooled testing for SARS-CoV-2.*

Dear Editors,

thank you for the possibility of sending a revised version of our revised manuscript 'Boosting test-efficiency by pooled testing for SARS-CoV-2'. Please find it enclosed with a slightly modified title that fits the PLOS ONE requirements.

We thank you for forwarding the constructive referee reports that we took into account very carefully for the revision. We have made a real effort and think that we could address all of their criticism and include their suggestions for a significant improvement of the manuscript. We are very happy with the corrections, additions and clarifications.

In a nutshell, the major points of the revision are: (1) The correction from Eq. (3) to conditional probabilities. (2) A discussion of limitations to pooling samples for group testing. (3) Additional references to relevant work pertinent to this context, and (4) a discussion of possible group size dependencies of false negative rates of pooled tests, including (5) an additional figure to demonstrate the effect of group size dependencies of false negative rates of pooled tests, and finally, (6) additional supporting information that includes the Matlab files and functions we used for producing the figures in the manuscript, and some detailed discussion of subtleties with regard to tests with replicates,

we attach a detailed point-by-point response to the two referees.

We hope that the manuscript can be accepted for publication in its present form.
Looking forward to hearing from you,

best regards,

Rudolf Hanel

Response to editors' comments:

1. Please ensure that your manuscript meets PLOS ONE's style requirements, including those for file naming.

Response: We have updated the manuscript with respect to those requirements. In particular we have removed section numbers.

2. In your Data Availability statement, you have not specified where the minimal data set underlying the results described in your manuscript can be found. PLOS defines a study's minimal data set as the underlying data used to reach the conclusions drawn in the manuscript and any additional data required to replicate the reported study findings in their entirety. All PLOS journals require that the minimal data set be made fully available. For more information about our data policy, please see http://journals.plos.org/plosone/s/data-availability.
Upon re-submitting your revised manuscript, please upload your study?s minimal underlying data set as either Supporting Information files or to a stable, public repository and include the relevant URLs, DOIs, or accession numbers within your revised cover letter. For a list of acceptable repositories, please see http://journals.plos.org/plosone/s/data-availability#loc-recommended-repositories. Any potentially identifying patient information must be fully anonymized.

Response: In order to adhere to PLOS ONE data availability policy, we make the code available in text form in the supplementing materials (SI). Since PLOS ONE supporting informations, according to the SI guidelines, accepts zipped files we directly include, besides the SI manuscript (pdf) also all Matlab m-files and functions that were used for calculations and creating figures in the manuscript.

General comment:

We added a whole paragraph in the manuscript discussing and referencing SARS-CoV-2 related work pertinent to group testing in order to support our work with available data, including a total of 15 additional citations.

Response to Referee 1:

1. Excellent and important practical manuscript. Citation which arrives at similar conclusion and provides direct experimental evidence :
   Lancet Infect Dis 2020, Published Online, April 28, 2020
   https://doi.org/10.1016/S1473-3099(20)30362-5
   might be cited

   A limitation which might be briefly discussed is the differential sensitivity and specificity of specific "PCR" tests which use one or two) target sequences, and there are small differences between viral target genes and the abundance of target RNA species. Efficiency of the specific methods used for RNA extraction and reverse transcription are variables that may effect sensitivity and ultimately limit

pooling.

Response: We thank the reviewer for this excellent suggestion. We have added the citation, and several others, together with a brief discussion of differential sensitivity and specificity in pooled testing in the introduction section of the manuscript. Moreover, we added and discuss Fig3 to demonstrate the effects of the false negative rate of the pooled test increasing with pool-size on the false negative measure we propose for pooled tests.

Response to Referee 2:

1. Is it reasonable to assume the false positive/negative rate does not change when switched from the regular testing to pooled testing? Does it apply to COVID-19 screening? It is hard to believe using a pool of size 32 or 64 can have the same false rates as testing the individuals one-by-one.

   Response: We thank the reviewer for pointing out this problem. We have added several references to work analysing this problem, e.g. by checking the differential sensitivity and specificity between individual testing and pool testing. We also added a paragraph in the introduction pointing out limiting factor to pooling, such as false negative rates increasing with group size due to dilution of the viral RNA, or the variability of viral load in samples resulting in the variability of cycle threshold values in RNA-RN-PCR protocols available for SARS-CoV-2. We also added a new figure (Fig3) to demonstrate the effect of false negative rates increasing with group size. Moreover, we point out in the manuscript that for typical current disease prevalence values our method would suggest group sizes of about 20, which is within the range of group sizes current literature on pooled RNA-RN-PCR tests believes to work for SARS-CoV-2 detection in pooled samples.

2. What is the choice of r in practice? How does it affect test efficiency? Following the majority rule, suppose r=5, then in practice, once you observed 3 of them were positive, there is no need to test the remaining 2 replicates. How does this affect the calculation of number of tests needed?

   Response: We thankfully incorporated this observation into the revised manuscript. For $r = 3$ we get an effective reduction to $r^* = 2.5$ and for $r = 5$ to $r^* = 4.375$ which makes the error of simply using r instead of r^*, i.e. $r/r^* \leq 1.2$ to be not severe and Q as computed in the paper serve as an upper bound for the number of expected tests per person (i.e. PPT= $1/Q$ is a lower bound for expected numbers of persons per test). That is the bounds go in the right direction, i.e. the true values are slightly more favourable than the bounds (for $r = 1$ one gets $r = r^* = 1$ and Q is exact). The practical choices for the number of replicas however turn out to be $r = 1$ (best gain PPT) or $r = 2$ (good gain but much better false negative control). More replicas than 2 do not improve control over false negatives, in

terms of the number of positives we may maximally miss (i.e. PTRF (former FNPT), not false negative rate, FNR, of the test) except maybe for extremely low infection levels.

3. Line 47 on Page 2, I believe it should be "If the pooled sample is declared positive, we test each individual in the group separately" because of the majority rule you proposed.

   Response: We have adapted the sentence accordingly..

4. The derivation of (3-5) in-explicitly assumed that given the true infection statues, the test results are mutually independent. Is this assumption supported by COVID-19 tests? If COVID-19 tests declare a sample as positive if the measured viral loads exceed a predetermined threshold, then this assumption does not hold.

   Response: Thank you for pointing this out to us. Eq. 3 in the manuscript treated replicas as independent tests. I am still surprised we did not realize this. The formula in the revised manuscript respects the conditional dependence on whether a pool in fact contains infected individuals or not. What was Eq. 3 now has become Eq. 2 and 3. And what was Eq. 2 and 4 are now Eq. 4 and 5. We also added a note in this paragraph that sensitivity/specificity of a group test may differ from sensitivity/specificity of testing individuals (what we also point out in the introduction; compare point 1). All figures have been recomputed accordingly and a third figure has been added to demonstrate the effects of pool-size dependent false negative rates.

5. There is no COVID-19 data supporting the methodology.

   Response: We have added a paragraph in the introduction which references works that discusses issues of pooling in the context of Covid-19, including the CDC notes on that topic, and work that points out limiting factors such as issues with viral load of individuals and samples, and how dilution affects false positives and cycle threshold values in PCR testing. The general message: Pooling works but one should not choose group sizes too large and possibly test a pool twice to control false negatives, which matches the results of our work, as the upper range of pool-sizes currently indicated by our method is around 20 persons per test, which is also the upper range pooling is typically used in praxis (due to the limitation of dilution).

6. Literature review is not sufficient. See Kim et al. (2009, Biometrics).

   Response: We have added this citation (and also the Kim-Hudgens, 2007 review paper). In total we have added 15 citations with relevance to group testing in general and group testing in the COVID-19 context in particular.

We hope that the referees are similarly happy with the improvement of the manuscript as we are. Thank you both for your efforts.
